# Psychoimmunological effects of dioscorea in ovariectomized rats: role of anxiety level

**DOI:** 10.1186/1744-859X-6-21

**Published:** 2007-08-10

**Authors:** Ying-Jui Ho, Ching-Fu Wang, Wen-Yu Hsu, Ting Tseng, Cheng-Chin Hsu, Mei-Ding Kao, Yuan-Feen Tsai

**Affiliations:** 1 School of Psychology, Chung Shan Medical University, No. 110, Sec. 1, Chien-Kuo N. Rd., Tai-Chung City 402, ROC, Taiwan; 2 School of Nutrition, Chung Shan Medical University, No. 110, Sec. 1, Chien-Kuo N. Rd., Tai-Chung City 402, ROC, Taiwan; 3 Department of Food and Nutrition, Providence University, No. 200 Chung-Chi Rd., Tai-Chung City 43301, ROC, Taiwan; 4 Department of Physiology, College of Medicine, National Taiwan University, No. 1, Sec. 1, Jen-Ai Rd., Taipei City 100, ROC, Taiwan

## Abstract

**Background:**

Anxiety levels in rats are correlated with interleukin-2 (IL-2) levels in the brain. The aim of the present study was to investigate the effects of dioscorea (wild yam), a Chinese medicine, on emotional behavior and IL-2 levels in the brain of ovariectomized (OVX) rats.

**Methods:**

One month after ovariectomy, female Wistar rats were screened in the elevated plus-maze (EPM) test to measure anxiety levels and divided into low anxiety (LA) and high anxiety (HA) groups, which were then given dioscorea (250, 750, or 1500 mg/kg/day) by oral gavage for 27 days and were tested in the EPM on day 23 of administration and in the forced swim test (FST) on days 24 and 25, then 3 days later, the brain was removed and IL-2 levels measured.

**Results:**

Compared to sham-operated rats, anxiety behavior in the EPM was increased in half of the OVX rats. After chronic dioscorea treatment, a decrease in anxiety and IL-2 levels was observed in the HA OVX rats. Despair behavior in the FST was inhibited by the highest dosage of dioscorea.

**Conclusion:**

These results show that OVX-induced anxiety and changes in neuroimmunological function in the cortex are reversed by dioscorea treatment. Furthermore, individual differences need to be taken into account when psychoneuroimmunological issues are measured and the EPM is a useful tool for determining anxiety levels when examining anxiety-related issues.

## Background

Anxiety and depression are major symptoms in postmenopausal women. Decreased blood levels of sex hormones are thought to be involved in these disorders [1], as postmenopausal syndrome is significantly improved by hormone replacement therapy, especially by a combined estrogen-progesterone regimen [2]. Interleukin-2 (IL-2) has recently been implicated as a modulator of neuronal function [3]. Pawlak et al have reported that IL-2 mRNA levels in the striatum and prefrontal cortex are relevant to emotional behavior in the elevated plus-maze (EPM) test [4,5] and that striatal microinjection of IL-2 causes an increase in the open arm time in the EPM test [6]. There is evidence that IL-2 is involved in various emotional behaviors [7] and that systemic administration of IL-2 results in anxiogenic activity [8]. In addition, IL-2/15Rβ knockout mice exhibit decreased levels of anxiety behavior in the EPM test compared to wild-type and heterozygote mice [9].

Systemic administration of sex hormones, for example estrogen and progesterone, modifies the affective behavior of ovariectomized (OVX) Long-Evans rats, decreasing anxiety, fear, and pain responses, through actions in certain brain areas [10]. Dioscorea (wild yam) has long been used as a Chinese medicine for improving gastrointestinal, sensory, memory, and sexual-related functions, and also hot flushes and frequency of urination in postmenopausal women. Animal studies have been used to evaluate its effects on osteoporosis [11], diabetes [12], and hyperlipidemia [13], which are very common in postmenopausal women, but, as far as we are aware, there have been no studies to date on the effect of dioscorea on behavior. Diosgenin, the main steroidal saponin in dioscorea [14,15], is used to manufacture steroidal hormones, such as progesterone, estrogen, testosterone, and cortisone [16,17], by *in vitro *chemical modification [18]; however, a recent study on menopausal animals indicated that sex hormone levels might not be affected by diosgenin treatment [19]. Furthermore, dietary supplementation with dioscorea does not affect dehydroepiandrosterone levels in the blood [20]. It has therefore been hypothesized that dioscorea, and/or diosgenin might not serve as a precursor of sex hormones *in vivo*, but affect menopausal symptoms by another mechanisms.

Recent studies indicated that dioscorea has an anti-inflammatory action both *in vivo *[21] and *in vitro*, decreasing the production of cytokines [22]. However, little is known about the role of IL-2 in menopausal syndrome. As IL-2 in the brain is involved in emotional behavior and its effects are area-dependent [3,23], it was of interest to examine the effects of dioscorea on IL-2 levels in the brain of menopausal animals.

OVX rats were used as a menopausal animal model because the changes in biochemical and physiological function seen in these animals are comparable with those in menopausal women [24], i.e., decreased levels of progesterone and estrogen [25], an increased risk of cardiovascular disease [26], and an enhanced rate of bone loss [27,28], as well as an increased anxiety level [29]. The time spent in the open arm during the EPM test is used to evaluate unconditioned avoidance behavior as a measure of anxiety [30,31], while immobility, a despair behavior, in the forced swim test (FST) is used to measure learned helplessness as a model of depression [32]. As far as we are aware, there are no published studies of the effect of dioscorea on anxiety and depression caused by OVX. In determining whether the responses of emotional behavior and IL-2 function in OVX rats to dioscorea treatment differed between low anxiety (LA) and high anxiety (HA) rats, we screened a group of OVX rats using the EPM test and divided them into LA and HA rats, then subjected both groups to chronic dioscorea treatment and measured their behavioral responses in the EPM and FST. The effects of dioscorea on IL-2 levels in brain tissue were also examined using an enzyme linked immunosorbent assay (ELISA).

## Methods

### Animals

Female Wistar rats (261 ± 4 g; n = 99; National Laboratory Animal Center, ROC) were used and housed in groups of five in acrylic cages (35 × 56 × 19 cm) in an animal room with a 12 h light/dark cycle (lights on at 07:00) with food and water provided *ad libitum*. Each animal was handled for 15 min/day on two consecutive days prior to the experiment. All experimental procedures were performed according to the NIH Guide for the Care and Use of Laboratory Animals and were approved by the Animal Care Committee of Chung Shan Medical University.

### General procedure

On day 28 after ovariectomy, a 10 min open field test was performed, followed by a 5 min EPM test on day 29. The animals were then divided into LA and HA rats and were given dioscorea (250, 750, or 1500 mg/kg/day) or vehicle (distilled water) by oral gavage for 27 days. On day 23 of dioscorea treatment, the EPM test was performed, followed by a FST on days 24 and 25 (see details of the schedule in Table 1). All behavioral tests were begun 2 h after start of lights-on and were performed before the dioscorea treatment for that day. The animals were weighed in the animal room, placed individually in a clean cage (25 × 41 × 19 cm), and transported to a dim observation room (28 lux) for behavioral testing. The test equipment was thoroughly cleaned using 20% alcohol followed by thorough drying before each rat was tested. The behavioral parameters of the open field test were analyzed using an automated computer program, while behavior in the EPM test and the FST was scored from videotapes.

**Table 1:** Schedule of treatment and behavioral tests in the present study

**Day**	**1**	**2–27**	**28**	**29**	**30–50**	**51**	**52**	**53**	**54–55**	**56**
**Treatment**	Ovariectomy			Dio	Dio	Dio	Dio	Dio	Dio	Killed
**Test**			Open field	EPM		EPM	FST day 1	FST day 2		
			(10 min)	(5 min)		(5 min)	(15 min)	(5 min)		

Animals underwent ovariectomy on day 1 and were killed on day 56. Dioscorea (Dio) was given daily by oral gavage from day 29 to day 55. EPM, elevated plus-maze test; FST, forced swim test.

### Ovariectomy

An aseptic surgical procedure was employed for all animals. The rats were anesthetized using ketamine (100 mg/kg, IM), then the dorsal part of the lumbar region was shaved, and the site cleaned with 75% ethanol followed by thorough scrubbing with 10% povidone iodine. A 2 cm incision was made in the skin through the musculature and peritoneum and the ovaries were retracted and removed. The wound was then closed using a 4-O sterile suture. Immediately after surgery, each rat was injected with penicillin-G procaine (0.2 ml, 20000 IU, IM), and the wound again cleaned with povidone iodine to reduce the chance of post-operative infection. The sham-operated group underwent the same surgical procedure except for the removal of the ovaries. After OVX, the rats were housed individually in plastic cages (25 × 41 × 19 cm) for about 10 days for recovery, then re-grouped in their home cages.

### Behavioral tests

#### Open field

The open field consisted of an acrylic box (40 × 40 × 40 cm). The movement distance of the rat during a 10 min observation was monitored using an automated activity monitoring system (Digiscan-16 Animal Activity Monitor System; model RXYZCM, Omnitech Electronics Columbus, OH, USA) [33].

#### EPM test

The EPM apparatus was made of plastic and consisted of two opposed open arms (50 × 10 cm), two opposed enclosed arms with no roof (50 × 10 × 40 cm), and an open square (10 × 10 cm) in the center, and was located 50 cm above the floor. Behavior in the EPM was observed for 5 min as described previously [34]. The following measures were analyzed from videotapes: (1) arm time: the time spent in open or enclosed arms, (2) arm entries: the number of entries into open or enclosed arms, and (3) arm activity: the number of time an animal crossed a virtual line that divided an arm into a proximal and a distal half. An entry into any of the compartments was defined as all four paws being placed in the compartment. The EPM test was performed twice in this study: once 4 weeks after ovariectomy and once on day 23 of dioscorea treatment. The open arm time in the first EPM test was used to screen individual anxiety levels and to establish high and low open arm responder groups of the same size.

#### Forced swim test

This test was carried out in a clear glass tank (25 × 25 × 60 cm) containing 39 cm of clean water at 26°C. The apparatus was cleaned thoroughly and the water changed between tests on different rats. A swimming test was performed on two consecutive days (15 min on day 1 and 5 min on day 2) and videotaped as described previously [35]. Immobility was measured from the videotapes and was defined as when the rats remained motionless or floating (including small limb movements to keep their heads above the water) [36]. To determine the effect of the FST on IL-2 levels, one-third of the rats were randomly assigned not to be subjected to the FST.

### Measurement of IL-2 levels

Three days after the FST, the rats were killed by exposure to CO_2 _and their brains immediately removed. The prefrontal cortex (the rostral part of the cortex, not including the forceps minor corpus callosum, about 12 mm anterior of the coronal plane passing through the interaural line, according to the atlas of Paxinos and Watson [37]) and the rest of the cerebral cortex (termed the "cerebral cortex" in this study) were dissected out on an ice-bath plate. The protein in the tissue was extracted by homogenizing the tissue in ice-cold lysis buffer (50 mM Tris-HCl, pH7.6, 0.5% NP-40, 150 mM NaCl, 1 mM EDTA, 10% glycerol containing protease inhibitors (1 μg/ml of aprotinin, 0.5 μg/ml of leupeptin, and 100 μg/ml of 4-(2-aminoethyl) benzenesulfonyl fluoride)). The homogenate was centrifuged at 2900 *g *for 15 min at 4°C (Hermle Z323 K centrifuge, Gosheimerstr, Germany), the supernatant re-centrifuged under the same conditions, and the final supernatant taken and its protein concentration measured using a Bio-Rad protein assay kit (Bio-Rad laboratories, CA, USA). A sample containing about 30–40 μg of protein was used to measure IL-2 levels using an ELISA kit with monoclonal anti-mouse IL-2 antibody (CytoSets™, BioSourse, CA, USA) according to the manufacturer's instructions. The color reaction was stopped after 30 min by addition of 2N H_2_SO_4 _and the optical density read at 450 nm within 30 min on an ELISA reader. The IL-2 levels were then calculated from a standard curve.

### Dioscorea

Dioscorea (D. L. alata. Var. purpurea (Roxb.) M. Pouch; Tainung No. 1 Shan-Yao) was purchased from Ming-Jean town, Nan Tao County, Taiwan. The yam tubers were cleaned, peeled, sliced into 1 cm wide slices, and boiled for 30 min to inhibit the browning reaction. Put the cooked sample, thereafter, to the moisture to around 10%, milled to a flour that passed through a 60 mesh sieve, and stored at -25°C until use. The dose of dioscorea was freshly prepared before use by adding double distilled water and mixing.

### Data analysis

As in our previous study [34], the OVX rats were ranked using the open arm time in the first EPM test, then assigned, using the median value, to two subgroups with high anxiety levels (34 animals with a shorter open arm time; HA rats) or low anxiety levels (34 animals with a longer open arm time; LA rats). These groups were used to examine the effect of dioscorea on behavior and IL-2 levels. Statistical testing was performed to compare within or between groups using *t*-tests for paired or unpaired data. Analysis of the effects of dioscorea was carried out by one-way analysis of variance (ANOVA), followed by a least-significant difference (LSD) post hoc test. All results are expressed as the mean ± SEM. The level of significance was defined as p < 0.05.

## Results

### Behavior after ovariectomy

Four weeks after ovariectomy, the open arm time of OVX rats in the EPM test was shorter than that of sham-operated rats (df = 97, *t *= 2.576, p = 0.012), whereas the enclosed arm time was longer in OVX rats than sham-operated rats (df = 97, *t *= 2.572, p = 0.012). Open arm activity, closed arm activity, and total arm activity were not different between OVX and sham-operated rats (Table 2). Furthermore, the movement distance in the open field test was not different between OVX and sham-operated rats (2644 ± 129 cm vs 2903 ± 132 cm).

**Table 2:** Behavior in the EPM test at 4 weeks after ovariectomy

		**OVX**		
		
	**Sham **(n = 31)	(n = 68)	LA subgroup (n = 34)	HA subgroup (n = 34)
OAT	50.6 ± 7.4	30.8 ± 4.0 ^#^	56.3 ± 4.6	5.3 ± 1.6 ^##^**
CAT	215.7 ± 9.5	241.1 ± 5.1 ^#^	211.2 ± 5.7	270.9 ± 4.2 ^##^**
OAA	6.0 ± 1.1	4.1 ± 0.7	7.6 ± 1.0	0.7 ± 0.3 ^##^**
CAA	21.2 ± 1.8	24.3 ± 1.1	21.6 ± 1.3	26.9 ± 1.6 ^#^*
TAA	27.2 ± 2.1	28.4 ± 1.2	29.2 ± 1.8	27.6 ± 1.7

LA, low anxiety; HA, high anxiety; OAT, open arm time; CAT, enclosed arm time; OAA, open arm activity; CAA, enclosed arm activity; TAA, total arm activity. #, p < 0.05; ##, p < 0.001, compared to the sham-operated group; *, p < 0.05; **, p < 0.001, compared to the LA subgroup. The data are expressed as the mean ± SEM.

The OVX rats were divided into HA and LA subgroups based on the median value of the OVX rats for the open arm time in the first EPM test [34]. These subgroups (each of 34 rats) had the following profiles: The open arm time and open arm activity were significantly lower in HA rats than in LA rats (both p values < 0.001), while the enclosed arm time and enclosed arm activity were significantly higher than in LA rats (both p values < 0.05). Total arm activity was not different between HA and LA rats. Interestingly, all of these values in LA rats were similar to those in sham-operated rats (Table 2).

### Behavior after dioscorea treatment

Dioscorea did not affect the behavior of sham-operated rats in the EPM test (Table 3), but significantly changed the EPM behavior of OVX rats (Table 4). In HA OVX rats, the open arm time and open arm activity were increased (both p values < 0.01) and the enclosed arm time and enclosed arm activity decreased (both p values < 0.05) after treatment with 750 mg/kg/day compared to before treatment. Similar effects were observed at the dosage of 1500 mg/kg/day (p value < 0.05), while the dosage of 250 mg/kg/day had no effect. Interestingly, dioscorea at 250 mg/kg/day, but not at the other two dosages, significantly decreased the open arm time and open arm activity of OVX LA rats (both p values < 0.05). None of the dosages of dioscorea affected the total arm activity in the EPM test.

**Table 3:** Effect of chronic dioscorea administration on the behavior of sham-operated rats in the EPM test

		0 mg/kg/day (n = 11)	250 mg/kg/day (n = 11)	750 mg/kg/day (n = 9)
OAT	Before	40.4 ± 11.5	57.7 ± 11.9	54.4 ± 16.3
	After	41.5 ± 10.2	58.7 ± 13.0	35.7 ± 9.9
CAT	Before	228.9 ± 16.7	206.0 ± 16.3	211.4 ± 17.4
	After	221.7 ± 15.0	174.3 ± 25.5	225.8 ± 13.1
OAA	Before	3.9 ± 1.3	7.7 ± 1.7	6.6 ± 2.5
	After	6.2 ± 1.5	8.2 ± 1.6	6.0 ± 2.1
CAA	Before	20.5 ± 2.6	22.3 ± 3.9	20.7 ± 3.0
	After	22.6 ± 2.4	18.4 ± 2.6	23.6 ± 1.6
TAA	Before	24.4 ± 3.4	30.0 ± 3.6	27.2 ± 4.0
	After	28.7 ± 2.6	26.6 ± 3.1	29.6 ± 2.5

OAT, open arm time; CAT, enclosed arm time; OAA, open arm activity; CAA, enclosed arm activity; TAA, total arm activity. The data are expressed as the mean ± SEM. "Before" and "after" are the results for the EPM test before, and on day 23 of, dioscorea treatment.

**Table 4:** Effect of chronic dioscorea administration on the behavior of OVX rats in the EPM test

		**LA rats**				**HA rats**			
			
		0 mg/kg/day (n = 8)	250 mg/kg/day (n = 10)	750 mg/kg/day (n = 9)	1 500 mg/kg/day (n = 7)	0 mg/kg/day (n = 7)	250 mg/kg/day (n = 8)	750 mg/kg/day (n = 9)	1 500 mg/kg/day (n = 10)
OAT	Before	59.3 ± 8.9	50.6 ± 6.5	57.5 ± 9.6	59.6 ± 14.5	5.1 ± 3.6	6.0 ± 3.3	6.5 ± 3.4	3.9 ± 2.7
	After	32.3 ± 11.4	23.2 ± 7.1 **	49.6 ± 9.5	37.6 ± 14.2	10.8 ± 5.8	17.2 ± 6.9	33.5 ± 8.4 **	21.3 ± 7.2 *
CAT	Before	205.5 ± 9.4	220.8 ± 8.8	209.3 ± 12.5	206.6 ± 16.8	277.0 ± 11.8	269.2 ± 7.5	261.8 ± 9.6	276.3 ± 5.3
	After	228.3 ± 19.4	247.0 ± 14.6	211.1 ± 15.6	224.2 ± 22.4	264.2 ± 10.7	264.9 ± 10.4	239.3 ± 13.9 *	239.6 ± 12.8 *
OAA	Before	8.0 ± 0.8	5.2 ± 0.9	9.7 ± 2.8	8.0 ± 2.8	0.6 ± 0.6	0.8 ± 0.5	0.9 ± 0.9	0.4 ± 0.3
	After	6.3 ± 1.9	2.8 ± 0.9 *	7.6 ± 1.9	5.4 ± 2.3	1.1 ± 0.9	2.5 ± 1.2	3.9 ± 1.3 **	1.8 ± 0.7
CAA	Before	21.8 ± 3.4	19.5 ± 1.3	24.0 ± 2.8	21.3 ± 3.4	27.1 ± 3.3	23.1 ± 3.4	28.4 ± 3.4	28.5 ± 3.1
	After	20.5 ± 3.5	22.1 ± 3.0	21.3 ± 2.4	21.1 ± 3.5	20.6 ± 1.9	18.1 ± 2.8	19.9 ± 2.6 *	24.9 ± 2.8
TAA	Before	29.8 ± 3.5	24.7 ± 1.7	33.7 ± 4.2	29.3 ± 5.3	27.7 ± 3.5	23.9 ± 3.5	29.3 ± 3.5	28.9 ± 3.0
	After	26.8 ± 4.0	24.9 ± 3.1	28.9 ± 3.0	26.6 ± 3.5	21.7 ± 2.0	20.6 ± 2.8	23.8 ± 2.5	26.7 ± 2.7

LA, low anxiety; HA, high anxiety; OAT, open arm time; CAT, enclosed arm time; OAA, open arm activity; CAA, enclosed arm activity; TAA, total arm activity. The data are expressed as the mean ± SEM. "Before" and "after" are the results for the EPM test before, and on day 23 of, dioscorea treatment. *, p < 0.05; **, p < 0.01, paired *t*-test, compared to the data before dioscorea treatment.

### Forced swim test

**Figure 1:**
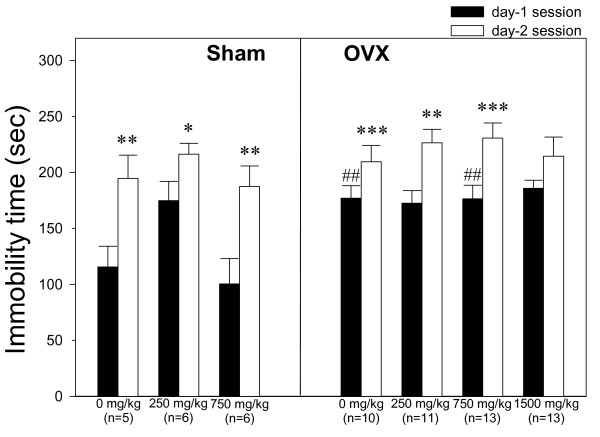
**Effects of chronic administration of dioscorea on immobility in the forced swim test**. Dioscorea (0, 250, 750, or 1 500 mg/kg/day) was given by oral gavage for 27 days and the rats were tested on days 24 and 25. The number of rats in each group is shown in parentheses below each group. * p < 0.05, ** p < 0.01, *** p < 0.001, compared to the data for the first session, paired *t*-test. ## p < 0.01, compared to sham-operated rats treated with the same dosage. The data are expressed as the mean ± SEM.

The immobility time during the first 5 min of the first FST in OVX rats treated with vehicle (distilled water) or 750 mg/kg dioscorea was significantly higher than that in sham-operated rats treated with the same dosage (both p values < 0.01, *t*-test). Learned helplessness was observed in all groups; the immobility time in the second FST session was significantly longer than that on the previous day (all pvalues < 0.05), except in the OVX group receiving 1500 mg/kg/day of dioscorea (Figure 1). Forced swimming behavior was not compared between HA and LA rats because there was no correlation between the immobility time in the first FST session and anxiety levels shown by the open arm time in the first EPM test. In addition, the number of animals in each group was too small to reach statistical power when the rats were divided into HA and LA subgroups (a third of the rats were not used in the FST).

### IL-2 levels

IL-2 levels in the brain areas analyzed were not influenced by the animals being subjected to the FST (data not shown), so the combined data for these two groups were used. As shown in Table 5, none of the dosages of dioscorea used affected IL-2 levels in the prefrontal cortex and cerebral cortex in sham-operated rats. IL-2 levels in the prefrontal cortex of vehicle-treated OVX LA rats were lower than those in vehicle-treated sham-operated rats (p < 0.05); however, this was reversed by treatment with dioscorea at the dosage of 250 mg/kg/day (p < 0.05), but not the other dosages. Levels in the cerebral cortex were unaffected by any dosage. In addition, all three dosages used in this study (250, 750, and 1500 mg/kg/day) significantly decreased IL-2 levels in the cerebral cortex of OVX HA rats compared to vehicle-treated OVX HA rats (p-values < 0.05), but had no effect on levels in the prefrontal cortex (Table 5).

**Table 5:** Effect of chronic dioscorea administration on IL-2 levels in the brain

				**OVX**							
				
	**Sham**			**LA rats**				**HA rats**			
			
	0 mg/kg (n = 10)	250 mg/kg (n = 11)	750 mg/kg (n = 9)	0 mg/kg (n = 8)	250 mg/kg (n = 10)	750 mg/kg (n = 9)	1 500 mg/kg (n = 7)	0 mg/kg (n = 7)	250 mg/kg (n = 7–8)	750 mg/kg (n = 9)	1 500 mg/kg (n = 10)
Prefrontal cortex	2.4 ± 0.2	2.7 ± 0.3	2.4 ± 0.2	1.7 ± 0.2#	2.5 ± 0.1*	2.2 ± 0.3	1.8 ± 0.3	1.9 ± 0.2	2.1 ± 0.2	2.3 ± 0.3	2.2 ± 0.2
Cerebral cortex	1.9 ± 0.4	1.8 ± 0.3	1.3 ± 0.2	1.6 ± 0.2	1.8 ± 0.3	2.1 ± 0.4	1.7 ± 0.2	2.7 ± 0.6	1.5 ± 0.2*	1.5 ± 0.2**	1.4 ± 0.2**

LA, low anxiety; HA, high anxiety. #, p < 0.05, compared to the untreated sham-operated rats; *, p < 0.05; **, p < 0.01, compared to the untreated rats in the same anxiety category. The data are expressed as the mean ± SEM. The units are pg/μg protein.

## Discussion

The present study showed that, at 1 month after ovariectomy, anxiety levels were highly increased in half of the rats. Chronic administration of dioscorea at dosages of 750 and 1500 mg/kg/day, but not at 250 mg/kg/day, had an anxiolytic activity in HA OVX rats, increasing the open arm time and decreasing the enclosed arm time in the EPM test. However, a lower dosage of dioscorea, 250 mg/kg/day, (but not the dosages of 750 and 1500) increased anxiety levels in LA OVX rats. These behavioral data are compatible with the data for IL-2 levels, which showed that IL-2 levels in the cerebral cortex of HA OVX rats were significantly decreased by all three dosages of dioscorea, while IL-2 levels in the prefrontal cortex of LA OVX rats were increased by dioscorea at 250 mg/kg/day. These effects of dioscorea were not due to non-specific effects on activity, as no effect was seen on total arm activity in the EPM test. In addition, learned helplessness in the FST was inhibited by dioscorea at 1500 mg/kg/day. The present data suggest that IL-2 in the brain could play a role in postmenopausal anxiety and could be involved in the mechanisms by which dioscorea decreases anxiety levels in HA OVX rats.

OVX rats are used as a menopausal animal model, as the changes in biochemical and physiological function are comparable with those seen in menopausal women [24], i.e. decreased levels of progesterone and estrogen [25], increased risk of cardiovascular disease [26], and enhanced rate of bone loss [27,28]. Although anxiety and depression are very common in menopausal women and elevation of anxiety levels has been reported in Long-Evans [38] and Wistar rats [29] after ovariectomy, the present study indicated that, compared to the sham-operated group, only half of OVX rats fell into the "high anxiety" group. This finding is compatible with results from clinical research, as anxiety is not seen in all postmenopausal women [39].

Dioscorea has long been used as a Chinese medicine for improving gastrointestinal, sensory, memory, and sexual-related functions. Several lines of evidence have demonstrated that it is effective in the treatment of osteoporosis [11], diabetes [12], and hyperlipidemia [13]; but there have not been any reports of its effect on behavior. Our data showed that oral administration of dioscorea decreased anxiety and depressive behavior in menopausal animals.

The sex hormone system could be involved in the behavioral effects of dioscorea. Decreased blood levels of sex hormone are thought to be involved in disorders after menopause [1], as postmenopausal syndrome is significantly improved by hormone replacement therapy, especially by a combined estrogen-progesterone regimen [2]. Diosgenin, the main steroidal saponin in dioscorea [14,15], is used to manufacture steroidal hormones, such as progesterone, estrogen, testosterone, and cortisone [16,17]. There are no reports on the exact mechanisms by which diosgenin is converted to other hormones *in vivo*, but a previous study showed that hypertrophy of the adrenal cortex in OVX animals was reversed towards control values after continuous supplementation with diosgenin [19]. Furthermore, the consumption of wild Mexican yam products containing diosgenin increases progesterone activity in the saliva [40], suggesting that the steroidal hormone system is affected.

IL-2 has recently been implicated as a modulator of neuronal function and emotional behavior [3]. IL-2 can influence neuronal activity [7], and an anxiogenic effect has been observed after the systemic administration of IL-2 [8]. The EPM is a widely used behavioral paradigm in the field of experimental anxiety research [41], and the values in this test are reported to correlate with anxiety-like and fear-motivated avoidance behavior [31,42]. During a typical EPM test, animals spend most of their time in the enclosed arms, rather than the open arms of the plus-maze, showing defensive behavior. Pawlak et al [6] reported that striatal microinjection of IL-2 affects emotional behavior in the EPM test. In addition, IL-2/15Rβ knockout mice exhibit decreased levels of anxiety-like behaviour in the EPM test compared to wild-type and heterozygote mice [9]. Chronic administration of IL-2 causes a reduction in exploration and approach to a novel stimulus [43], indicating a correlation between IL-2 and defensive behavior. In the present study, decreased avoidance of the open arm of the EPM correlated with lowered levels of IL-2 in the cerebral cortex in HA OVX rats treated with dioscorea. Furthermore, a decrease in open arm time and an increase in IL-2 level in the prefrontal cortex of LA OVX rats were seen after treatment with dioscorea at 250 mg/kg/day.

A previous study indicated that intracerebroventricular administration of IL-1beta and tumor necrosis factor-α provokes an anxiogenic response in the EPM test without affecting neurotransmitter concentrations in the amygdala [23]. However, the dopaminergic system in the striatum is reported to be sensitive to modulation by IL-2 [3]. The present study showed that the anxiogenic effects of 250mg/kg/day of dioscorea in LA OVX rats were accompanied by an increase in IL-2 levels in the prefrontal cortex, while anxiolytic activity in HA OVX rats was accompanied by a decrease in IL-2 levels in the cerebral cortex. These data support the view that the function of cytokines is area-specific [3,5]. As the amygdala is correlated with the pathophysiology of anxiety, the function of IL-2 in this area deserves further study.

The immobility time in the first FST session was higher in OVX rats than in sham-operated rats, suggesting that the basal level of despair behavior was higher in OVX rats. In addition, only the highest dosage of dioscorea blocked learned helplessness, while the anxiolytic effects were observed at lower dosages, showing that the biological basis of anxiety and depression is not identical [34,35]. Furthermore, the effects of dioscorea on behavior and IL-2 levels were dependent on the anxiety levels of the OVX rats and had task-dependent behavioral consequences, indicating that cytokine responses to treatment might be involved in the individual differences in anxiety levels.

## Conclusion

Compared to the sham-operated group, anxiety levels were higher in 50% of the OVX rats. The anxiolytic activity of chronic dioscorea treatment correlated with a decrease in IL-2 levels in the cerebral cortex of HA OVX rats. In contrast, the anxiogenic effect of dioscorea in LA OVX rats was accompanied by an increase in IL-2 levels in the prefrontal cortex. In addition, learned helplessness in the FST was decreased by the highest dosage of dioscorea. The present results provide a new insight into the pathophysiological role of IL-2 in postmenopausal anxiety. IL-2 could be involved in the mechanisms underlying the behavioral effects of dioscorea.

## Competing interests

The author(s) declare that they have no competing interests.

## Authors' contributions

YJH conceived, designed, and coordinated the study, participated in the data collection, performed the statistical analysis, and drafted the manuscript. YFT revised the manuscript critically for important intellectual content. The other authors participated in data collection. All authors read and approved the final manuscript.

## References

[B1] Davidson JM (1985). Sexual behavior and its relationship to ovarian hormones in the menopause. Maturitas.

[B2] Linzmayer L, Semlitsch HV, Saletu B, Bock G, Saletu-Zyhlarz G, Zoghlami A, Gruber D, Metka M, Huber J, Oettel M, Graser T, Grunberger J (2001). Double-blind, placebo-controlled psychometric studies on the effects of a combined estrogen-progestin regimen versus estrogen alone on performance, mood and personality of menopausal syndrome patients. Arzneimittelforschung.

[B3] Petitto JM, McCarthy DB, Rinker CM, Huang Z, Getty T (1997). Modulation of behavioral and neurochemical measures of forebrain dopamine function in mice by species-specific interleukin-2. J Neuroimmunol.

[B4] Pawlak CR, Ho YJ, Schwarting RK, Bauhofer A (2003). Relationship between striatal levels of interleukin-2 mRNA and plus-maze behaviour in the rat. Neurosci Lett.

[B5] Pawlak CR, Schwarting RK, Bauhofer A (2005). Cytokine mRNA levels in brain and peripheral tissues of the rat: relationships with plus-maze behavior. Brain Res Mol Brain Res.

[B6] Pawlak CR, Schwarting RK (2006). Striatal microinjections of interleukin-2 and rat behaviour in the elevated plus-maze. Behav Brain Res.

[B7] Hanisch UK, Ader R, Felten DL, Cohen N (2001). Effects of Interleukin-2 and interferons on the nervous system. Psychoneuroimmunology.

[B8] Koh KB, Lee Y (2004). Reduced anxiety level by therapeutic interventions and cell-mediated immunity in panic disorder patients. Psychother Psychosom.

[B9] Petitto JM, Huang Z, Hartemink DA, Beck R (2002). IL-2/15 receptor-beta gene deletion alters neurobehavioral performance. Brain Res.

[B10] Frye CA, Walf AA (2004). Estrogen and/or progesterone administered systemically or to the amygdala can have anxiety-, fear-, and pain-reducing effects in ovariectomized rats. Behav Neurosci.

[B11] Yin J, Kouda K, Tezuka Y, Tran QL, Miyahara T, Chen Y, Kadota S (2003). Steroidal glycosides from the rhizomes of *Dioscorea spongiosa *. J Nat Prod.

[B12] Iwu MM, Okunji CO, Ohiaeri GO, Akah P, Corley D, Tempesta MS (1990). Hypoglycaemic activity of dioscoretine from tubers of *Dioscorea dumetorum *in normal and alloxan diabetic rabbits. Planta Med.

[B13] Chen H, Wang C, Chang CT, Wang T (2003). Effects of Taiwanese yam (*Dioscorea japonica *Thunb var. pseudojaponica Yamamoto) on upper gut function and lipid metabolism in Balb/c mice. Nutrition.

[B14] Marker RE, Wagner RB, Ulshafer PR, Wittbecker EL (1943). Sterols. CLVII. Sapogenins. LXIX. Isolation and structures of thirteen new steroidal sapogenins. New sources for known sapogenins. J Am Chem Soc.

[B15] Marker RE, Turner DL, Ulshafer PR (1940). Sterols. CIV. Diosgenin from certain American plants. J Am Chem Soc.

[B16] Marker RE (1940). Sterols. CV. The preparation of testosterone and related compounds from sarsasapogenin and diosgenin. J Am Chem Soc.

[B17] Rosenkranz G, Djerassi C, Yashin R, Pataki J (1951). Cortical hormones from allsteroids; synthesis of cortisone from Reichsteen's compound D. Nature.

[B18] Marker RE (1940). Dioscoreaceae. J Am Chem Soc.

[B19] Benghuzzi H, Tucci M, Eckie R, Hughes J (2003). The effects of sustained delivery of diosgenin on the adrenal gland of female rats. Biomed Sci Instrum.

[B20] Araghiniknam M, Chung S, Nelson-White T, Eskelson C, Watson RR (1996). Antioxidant activity of dioscorea and dehydroepiandrosterone (DHEA) in older humans. Life Sci.

[B21] Lee SC, Tsai CC, Chen JC, Lin CC, Hu ML, Lu S (2002). The evaluation of reno- and hepatoprotective effects of huai-shan-yao (Rhizome Dioscoreae). Am J Chin Med.

[B22] Kim MJ, Kim HN, Kang KS, Baek NI, Kim DK, Kim YS, Jeon BH, Kim SH (2004). Methanol extract of Dioscoreae Rhizoma inhibits pro-inflammatory cytokines and mediators in the synoviocytes of rheumatoid arthritis. Int Immunopharmacol.

[B23] Connor TJ, Song C, Leonard BE, Merali Z, Anisman H (1998). An assessment of the effects of central interleukin-1beta, -2, -6, and tumor necrosis factor-alpha administration on some behavioural, neurochemical, endocrine and immune parameters in the rat. Neuroscience.

[B24] Bosse R, Di Paolo T (1995). Dopamine and GABAA receptor imbalance after ovariectomy in rats: model of menopause. J Psychiatry Neurosci.

[B25] Erb RE, Gomes WR, Randel RD, Estergreen VL, Frost OL (1968). Effect of ovariectomy on concentration of progesterone in blood plasma and urinary estrogen excretion rate in the pregnant bovine. J Dairy Sci.

[B26] Sharkey LC, Holycross BJ, Park S, Shiry LJ, Hoepf TM, McCune SA, Radin MJ (1999). Effect of ovariectomy and estrogen replacement on cardiovascular disease in heart failure-prone SHHF/Mcc- fa cp rats. J Mol Cell Cardiol.

[B27] Katase K, Kato T, Hirai Y, Hasumi K, Chen JT (2001). Effects of ipriflavone on bone loss following a bilateral ovariectomy and menopause: a randomized placebo-controlled study. Calcif Tissue Int.

[B28] Higdon K, Scott A, Tucci M, Benghuzzi H, Tsao A, Puckett A, Cason Z, Hughes J (2001). The use of estrogen, DHEA, and diosgenin in a sustained delivery setting as a novel treatment approach for osteoporosis in the ovariectomized adult rat model. Biomed Sci Instrum.

[B29] Fernandez-Guasti A, Ferreira A, Picazo O (2001). Diazepam, but not buspirone, induces similar anxiolytic-like actions in lactating and ovariectomized Wistar rats. Pharmacol Biochem Behav.

[B30] Blanchard DC, Blanchard RJ, Tom P, Rodgers RJ (1990). Diazepam changes risk assessment in an anxiety/defense test battery. Psychopharmacology (Berl).

[B31] Pellow S, Chopin P, File SE, Briley M (1985). Validation of open:closed arm entries in an elevated plus-maze as a measure of anxiety in the rat. J Neurosci Methods.

[B32] Porsolt RD, Le Pichon M, Jalfre M (1977). Depression: a new animal model sensitive to antidepressant treatments. Nature.

[B33] Ho YJ, Chang YC, Liu TM, Tai MY, Wong CS, Tsai YF (2000). Striatal glutamate release during novelty exposure-induced hyperactivity in olfactory bulbectomized rats. Neurosci Lett.

[B34] Ho YJ, Eichendorff J, Schwarting RK (2002). Individual response profiles of male Wistar rats in animal models for anxiety and depression. Behav Brain Res.

[B35] Ho YJ, Hsu LS, Wang CF, Hsu WY, Lai TJ, Hsu CC, Tsai YF (2005). Behavioral effects of d-cycloserine in rats: the role of anxiety level. Brain Res.

[B36] Armario A, Gil M, Marti J, Pol O, Balasch J (1991). Influence of various acute stressors on the activity of adult male rats in a holeboard and in the forced swim test. Pharmacol Biochem Behav.

[B37] Paxinos G, Watson C (1986). The Rat Brain in Stereotaxic Coordinates.

[B38] Zimmerberg B, Farley MJ (1993). Sex differences in anxiety behavior in rats: role of gonadal hormones. Physiol Behav.

[B39] Sagsoz N, Oguzturk O, Bayram M, Kamaci M (2001). Anxiety and depression before and after the menopause. Arch Gynecol Obstet.

[B40] Zava DT, Dollbaum CM, Blen M (1998). Estrogen and progestin bioactivity of foods, herbs, and spices. Proc Soc Exp Biol Med.

[B41] Rodgers RJ, Cole JC, Cooper SJ, Hendrie CA (1995). The elevated plus-maze: pharmacology, methodology and ethology. Ethology and psychopharmacology.

[B42] Handley SL, McBlane JW (1993). An assessment of the elevated X-maze for studying anxiety and anxiety-modulating drugs. J Pharmacol Toxicol Methods.

[B43] Lacosta S, Merali Z, Anisman H (1999). Influence of acute and repeated interleukin-2 administration on spatial learning, locomotor activity, exploratory behaviors, and anxiety. Behav Neurosci.

